# Awareness on obstetric fistula and associated factors among women health development army, in the South Gondar zone, Amhara region, Ethiopia: A cross sectional study

**DOI:** 10.1002/hsr2.70141

**Published:** 2024-10-14

**Authors:** Bilikew Addimasu, Dabere Nigatu, Zemenu S. Yadita, Mekonnen Melkie

**Affiliations:** ^1^ South‐Gondar Health Office Amhara Regional Health Bureau Bahir Dar Amhara Region Ethiopia; ^2^ Department of Reproductive Health and Population Studies College of Medicine and Health Science, Bahir Dar University Bahir Dar Ethiopia

**Keywords:** awareness, Ethiopia, obstetric fistula, South Gondar, women health development army

## Abstract

**Background and Aims:**

Obstetric fistula is a severe birth injury that profoundly impacts women's health, social well‐being, economic stability, and psychological state. While it can be prevented with timely emergency obstetric care, a significant barrier in many developing regions, including the study area, is the lack of awareness among women. This study aimed to evaluate the level of awareness about obstetric fistula and the factors influencing it among the Women Health Development Army in the South Gondar zone of the Amhara region, Ethiopia, in 2020.

**Methods:**

A community‐based cross‐sectional study was done from July to December 2020, involving 633 women. Participants were selected using a multistage stratified sampling method. Data were collected using a pretested, interviewer‐administered structured questionnaire. Data entry was performed using EpiData version 3.1, and analysis was conducted with SPSS version 23.0. Multivariable logistic regression models were employed to identify influencing factors, with a *p* < 0.05 considered statistically significant.

**Results:**

This study found that 55.3% (95% CI, 51.6%–57.2%) of study participants had a good awareness of obstetrics fistula. Significantly associated factors include; no history of ANC (AOR = 0.52: 95% CI, 0.31, 0.86), living >30 min away from a health facility (AOR = 0.43: 95% CI, 0.23, 0.81), no history of abortion (AOR = 0.37: 95% CI, 0.19, 0.72), Injectable and implant users (AOR = 3.60: 95% CI, 1.98, 6.53), having four to six pregnancy (AOR = 1.87: 95% CI, 1.22, 2.87) and lack of communication material in the house (AOR = 0.19: 95% CI, 0.10, 0.37).

**Conclusions:**

This study found that 55.3% of women have a good awareness of obstetrics fistula. ANC follow‐up history, abortion history, type of family planning, type of communication, number of pregnancies, availability of health facility, and distance of health facility were significantly associated with awareness of obstetric fistula. Hence, local and national efforts should be made to enhance access to information at home and basic maternal health services including antenatal care.

## 1 INTRODUCTION

Obstetric fistula is a serious birth injury resulting from prolonged compression of the birth canal tissues, bladder base, urethra, or sometimes the rectum by the presenting fetal part.^1^, ^2^ This condition has a profound impact on the social, economic, and psychological well‐being of affected women. The associated stigma, deep sense of loss, and diminished dignity and identity can significantly undermine their quality of life. It has a devastating social, economic, and psychological effect on the health and well‐being of the affected women. The stigma, deep sense of loss, and loss of dignity and identity associated with fistula have a negative impact on quality of life.^3^, ^4^

Every year, approximately two million women worldwide suffer from untreated fistulas. Annually, between 50,000 and 100,000 new cases of obstetric fistula are diagnosed. In sub‐Saharan Africa, approximately 1000 women are affected by this condition.^5^, ^6^ Approximately two million women in sub‐Saharan Africa and Asia are living with untreated fistula. This condition is both preventable and treatable. Despite the establishment of various initiatives aimed at preventing and repairing fistulas in recent years, many women face delays in seeking treatment. Factors contributing to these delays include a lack of awareness about the possibility of repair, insufficient resources to access care, and obstacles such as shortages of medical personnel or facilities and inadequate quality of care.^7^, ^8^, ^9^, ^10^

Awareness of obstetric fistula involves understanding its symptoms, prevention strategies, and risk factors.^11^ A common obstacle to seeking treatment for fistula is a lack of awareness. Many women affected by obstetric fistula are unaware of what the condition is, whether it can be treated, or where to seek treatment. Additionally, both the women with fistulas and their communities may have misconceptions about the causes of the condition.^12^, ^13^

While obstetric fistula has been eradicated in high‐income countries, it continues to be a widespread and debilitating issue in many developing regions. In these areas, women often face immediate effects such as leakage of urine, feces, or both.^14^, ^15^ Awareness of obstetric fistula is still low in many developing countries, including Ethiopia (40%), where it is not uncommon.^11^ A systematic review found that 0.29% pooled prevalence of obstetric fistula; separated by region the highest prevalence was in sub‐Saharan Africa and South Asia, 1.60%. A study in Egypt, has found that showed that, three quarters of the studied pregnant women had in adequate level of total knowledge regarding obstetric fistula. One in 10 women proposed that suicide might be a solution to fistula, while one‐third believed that women with the condition should be isolated.^15^, ^16^

In Ethiopia, however, obstetric fistulas remain one of the factors contributing to maternal illness and death, as the health care system is still not well developed. The nation's young, small, and illiterate girls and women who live in the remote parts of the country are becoming the victims of this preventable and curable obstetric complication.^14^, ^17^ The EDHS 2016 data from Ethiopia indicates that only 39% of women aged 15–49 have heard of obstetric fistula. Additionally, fewer than 1% of women report having personally experienced the condition.^18^ The report also reveals that the prevalence of obstetric fistula is highest in certain regions and varies across the country. Tigray (1.6%) and the Southern Nations, Nationalities, and People's Region (SNNPR) (1.5%) have the highest rates of obstetric fistula, with Oromia following closely at 1.2%.^18^ Among reproductive‐age women who have heard of obstetric fistula, awareness is lowest in the Oromia region at 28.6%, compared to higher awareness levels in the Tigray and Amhara regions, which are 65.8% and 45%, respectively.^15^

The country's rural healthcare coverage remains deplorable. The efforts of health extension workers (HEWs) have been hindered by the extensive catchment areas and widely scattered populations they serve. Recent health reform initiatives have aimed at enhancing local primary health care (PHC) systems Due to these, Ethiopia launched a new initiative in 2010/2011, comprising community volunteers known as the “Women Health Development Army” (WHDA).^19^, ^20^ Efforts to improve women's health are crucial, particularly in less developed countries, to address the growing demand for healthcare services and the shortage of formal healthcare providers. It is understood that solutions cannot be achieved solely from the standpoint of service providers and program planners.^19^, ^20^

There is community‐based evidence of awareness of obstetric fistula and associated factors in Ethiopia, particularly in the study area, including the women's health and development army. Hence, this study has assessed the magnitude and factors associated with awareness of obstetric fistula among women in the health and development army in the South Gondar zone, Amhara region, Ethiopia, in 2020.

## 2 METHODOLOGY

### 2.1 Study design, and area

A community‐based cross‐sectional study was carried out from November to December 2020 in the Estie District of the South Gondar zone in the Amhara region of northwest Ethiopia. The district is served by 16 health facilities, including 1 primary hospital, 11 health centers, and 3 private clinics. It comprises 40 kebele (small administrative units), and is home to approximately 12,775 members of the women's health development army.^21^

### 2.2 Population

All women's health development army in the South Gondar zone, Amhara region were the source populations. While the study populations were all randomly selected women health development armies residing in the randomly selected kebeles.

#### 2.2.1 Inclusion criteria

All members of the Women's Health Development Army (WHDA) who self‐reported having no history of obstetric fistula were included.

#### 2.2.2 Exclusion criteria

Women health development army who were severely ill during data collection were excluded.

### 2.3 Sample size

The sample size for this study was calculated using a single population proportion formula. The calculation was based on several assumptions: a 50% awareness rate of obstetric fistula, derived from a study in the Oromia region of Ethiopia,^22^ a 95% confidence interval (CI), a 5% margin of error, a 10% nonresponse rate, and a design effect of 1.5. Taking these assumptions into account, the final sample size was determined to be 633.

### 2.4 Sampling procedure

A multistage stratified sampling approach was employed for the study. The Estie district was chosen through a lottery method, and out of its 40 kebeles, 8 were selected using the same method. The sample size was proportionally distributed among these selected kebeles. For each kebele, a list of eligible women's health development army members was compiled from registration records, with assistance from kebele administration and health posts. Subsequently, a computer‐generated simple random sampling technique was used to select participants from each kebele.

### 2.5 Variable

The outcome variable was “Awareness on obstetric fistula” measured as a dichotomous variable. The independent variables include; sociodemographic variables, obstetric and reproductive variables, health institution related variables, and media exposure.

### 2.6 Data collection tools

A structured, interviewer‐administered questionnaire was employed to gather information from study participants. This tool was adapted from existing literature.^11^, ^22^ The questionnaire was initially prepared in English, then translated into the local language (Amharic), and subsequently back‐translated into English to ensure consistency. The tool includes the following sections; sociodemographic characteristics, obstetric and reproductive factors, and other factors of awareness of obstetric fistula. Four diploma nurses were hired as data collectors, and two bachelor's degree nurses were recruited as supervisors. To ensure data quality, the questionnaires were carefully designed and pretested on 5% of the sample from nonselected women's health development army members in the district. A 1‐day training session was conducted to explain the study's objectives, data collection methods, and how to interact with participants during interviews. Supervisors and the principal investigator reviewed each collected questionnaire daily to ensure completeness.

### 2.7 Measurement

#### 2.7.1 Obstetric fistula

An obstetric fistula is a passageway that forms between the vagina and the urinary tract (including the ureter, urethra, and bladder) or the rectum, causing continuous leakage of urine, feces, or both.^23^

#### 2.7.2 Awareness

Conscious or informed about obstetric fistula, predisposing factors, and symptoms of obstetric fistula. In this study, it was assessed with 24 questions.

- Good Awareness: Those respondents who scored greater or equal to the mean value questions asked 24 awareness‐related questions about obstetric fistula.^24^
- Poor Awareness: Those respondents who scored less than the mean value of the question related to awareness of obstetric fistula.^24^

### 2.8 Data processing and analysis

The collected data was entered and cleaned using EPI Data version 3.1 and then exported to SPSS version 23 for analysis. Descriptive statistics, including percentages, means, and standard deviations, were used to present demographic data and the extent of awareness about obstetric fistula. Binary logistic regression was applied to identify factors associated with awareness of obstetric fistula. Bi‐variable analysis was performed to pinpoint variables with a *p* value of 0.2 or less, which were then included in multivariable logistic regression models to account for potential confounders. Statistical significance was determined with a *p* < 0.05, and adjusted odds ratios were calculated with a 95% confidence interval. The model's goodness of fit was assessed using the Hosmer–Lemeshow test (*p* = 0.505), and multicollinearity was evaluated using the variance inflation factor (VIF).

## 3 RESULTS

### 3.1 Sociodemographic characteristics of women and their husbands

Among the total of 633 sampled women, 609 participated in the study giving a response rate of 96.2%. Of all study participants, 445 (73.1%) of them were aged between ranges of 25–49 years old. The highest proportion of women 532 (87.4%) were Orthodox Christians, and nearly half 300 (49.3%) of the participants were illiterate. Concerning the educational status of women's husbands, 246 (40.4%) were illiterate and 217 (35.6%) attended elementary and above. Four hundred and ninety‐nine (81.9%) participants were housewives and 350 (57.5%) participants' husbands were farmers. But, 42 (6.9%) mothers and 48 (7.9%) participants' husbands were daily laborers. Five hundred and one (82.3%) of women health development army were married and living together with their husbands (Table 1).

**Table 1:** Sociodemographic characteristics of WHDA in the Estie District, South‐Gondar, Amhara Region, Ethiopia, 2021.

Variables (*n* = 609)	Categories	Frequency	Percentage
Age	15–24 years	97	15.9
	25–49 years	445	73.1
	Above 49 years	67	11
Religion	Orthodox Christian	532	87.4
	Muslims	77	12.6
Education of women	Cannot read and write	300	49.3
	Can read or write	162	26.6
	Primary and above	147	24.1
Occupation of women	House wife	499	81.9
	Farmer	65	10.7
	Merchant	42	6.9
	Other^a^	3	.5
Marital status of women	Married	501	82.3
	Single	38	6.2
	Divorced	38	6.2
	Separated	32	5.3
Education level of husband	Cannot read and write	203	40.4
	Elementary and above	178	35.6
	Can read and write	121	24.0
Occupation of the husband	Farmer	288	57.5
	Merchant	138	27.6
	Daily laborer	40	7.9
	Other	35	7.1
Communication material Avail in the house	Radio	255	41.9
	Television	73	12.0
	Mobile	179	29.4
	None	87	14.3
	Other	15	2.5
Time to reach to the health facility	<30 min	134	22.0
	≥30 min	472	77.5

^a^ Government or private employee and daily laborer.

### 3.2 Obstetric and health facility related factors

Five hundred and eighty‐six (96.2%) of the women health development army had a history of pregnancy. Of this 379 (64.7%) of them had one to three pregnancy history. Of those women who had a history of pregnancy, 79 (13%) of them had a history of abortion and 480 (78.8%) of them had a history of antenatal care visits for their last pregnancy. Six hundred and two (98.9%) of the participants had a history of modern FP methods use. Among family planning users 410 (68%) used injectable family planning methods. The majority of the study participants 564 (92.6%) reported the availability of the health facility in their kebeles. Two hundred and fourteen (35.1%) of participants got information regarding obstetric fistula from a health facility. Five hundred and seventy‐nine (95.2%) respondents had no training history in obstetric fistula (Table 2).

**Table 2:** Obstetric and health facility related characteristics of WHDA in the Estie District, South‐Gondar, Amhara Region, Ethiopia, 2021.

Variables (*n* = 609)	Categories	Frequency	Percentage
History of pregnancy	Yes	586	96.2
	No	23	3.8
History of abortion	Yes	79	13.0
	No	529	87.0
Number of pregnancy	1–3	379	64.7
	4–6	177	30.2
	7 and above	30	5.1
Family planning history	Yes	602	98.9
	No	7	1.1
Type of family planning method	Pills	36	5.9
	Injectable	410	68.0
	Implants	39	6.4
	Injectable and implants	117	19.2
Availability of nearer health facility	Yes	564	92.6
	No	45	7.4
Time to reach the health facility	≤30 min	135	22.2
	>30 min	474	77.9
Communication material avail in their house	Radio	255	41.9
	Television	73	12.0
	Magazine	15	2.4
	Mobile	179	29.4
	None	87	14.3
Training history about obstetric fistula	Yes	29	4.8
	No	579	95.2
Source of information about obstetric fistula	Health facility	214	35.1
	Family or friends	115	18.9
	Radio or television	26	4.3

### 3.3 Awareness toward obstetric fistula

Over half (57.5%) of women's reported that they heard about obstetric fistula. Of these, 35% of participants got information from health facilities about obstetric fistula. Among all the study participants, 55.3% (with a 95% confidence interval of 51.7%–57.2%) had a good level of awareness about obstetric fistula, while the remaining 44.7% had poor awareness (Table 3).

**Table 3:** Awareness of WHDA toward obstetric fistula in the Estie district, the South‐Gondar zone, Amhara region, Ethiopia, 2021.

Variables	Categories	Frequency	Percentage
Ever heard about obstetric fistula	Yes	350	57.5
	No	259	42.5
Awareness on symptoms of obstetric fistula	Unable to control urine	317	52.1
	Unable to control faces	122	20.0
	Irritation of genital area	214	35.1
	Unable to control flatus	122	20.0
	Abnormal vaginal discharge	226	37.1
	Recurrent urinary tract infection	215	35.3
Awareness on prevention of obstetric fistula	Health facility delivery	340	55.8
	Prevention of early marriage	314	51.6
	Safe abortion	307	50.4
	Avoid FGM	178	29.2
Overall awareness toward obstetric fistula	Good	337	55.3
	Poor	272	44.7

### 3.4 Factors associated with awareness toward obstetric fistula

In the bivariate analysis educational status, husband's occupation, husband's educational level, pregnancy history, number of pregnancies, history of antenatal care (ANC), abortion history, type of family planning, type of communication material avail in the house, availability of near health facility and time to reach the health facility were found to be candidate variables for multivariable logistic regression analysis with a *p* < 0.20. In the multivariable logistic regression analysis history of antenatal care, abortion history, type of family planning, type of communication material avail, number of pregnancies, availability of near health facility, and time to reach the health facility were significantly associated with awareness of obstetric fistula.

The result showed a significant association between a history of Antenatal care follow‐up and the odds of having good awareness. Those who had no ANC follow‐up were 48% less likely to have good awareness about obstetrics fistula than their counterparts (AOR = 0.52; 95% CI, 0.31, 0.86). Respondents who lived more than 30 min away from a health facility were 57% less likely to have good awareness compared to those living closer (AOR = 0.43; 95% CI, 0.23–0.81). Those without health facilities within their Kebesle were 67% less likely to have good awareness than their counterparts (AOR = 0.33; 95% CI, 0.15–0.71). Members of the Women's Health Development Army with no history of abortion were 62% less likely to have good awareness compared to those with a history of abortion (AOR = 0.37; 95% CI, 0.19–0.72). Women using both injectable and implant family planning methods were more than three times as likely to have good awareness compared to those using only injectable methods (AOR = 3.60; 95% CI, 1.98–6.53). Women with a history of four to six pregnancies were nearly twice as likely to have good awareness compared to those with fewer than three pregnancies (AOR = 1.87; 95% CI, 1.22–2.87). Additionally, those without communication materials in the house were 80% less likely to have good awareness compared to radio users (AOR = 0.19; 95% CI, 0.10–0.37) (Table 4).

**Table 4:** Multivariable logistic regression analysis of factors associated with awareness toward obstetric fistula among WHDA in the Estie district, the South‐Gondar zone, Amhara region, Ethiopia, 2021.

Variables	Categories	Awareness toward obstetric fistula		AOR^a^ (95%^b^ CI)	*p* Value
		Good	Poor		
ANC follow‐up	Yes	293 (61.0%)	187 (39.0%)	1	
	No	36 (33.6%)	71 (66.4%)	0.52 (0.31, 0.86)*	0.01
Abortion history	Yes	63 (79.7%)	16 (20.3%)	1	
	No	267 (52.4%)	243 (47.6%)	0.37 (0.19, 0.72)*	0.03
Number of pregnancy	1–3	197 (50.0%)	197 (50.0%)	1	
	4–6	125 (67.9%)	59 (32.1%)	1.87 (1.22, 2.87)*	0.04
	7 and above	15 (48.4%)	16 (51.6%)	1.37 (0.24, 7.83)	0.71
Type of family planning method	Inject able	200 (48.3%)	214 (51.7%)	1	
	Implants	23 (59.0%)	16 (41.0%)	1.21 (0.56, 2.62)	0.61
	Injectable and implants	96 (82.8%)	20 (17.2%)	3.60 (1.98, 6.53)*	<0.01
	Others^c^	18 (45.0%)	22 (55.0%)	0.80 (0.36, 1.80)	0.06
Communication material avail	Radio	150 (58.8%)	105 (41.2%)	1	
	Television	64 (87.7%)	9 (12.3%)	1.80 (0.73, 4.44)	0.19
	Mobile	92 (51.4%)	87 (48.6%)	0.73 (0.47, 1.13)	0.16
	None	20 (23.0%)	67 (77.0%)	0.19 (0.10, 0.37)*	<0.01
	Other	11 (73.3%)	4 (26.7%)	1.72 (0.49, 5.99)	0.39
Availability of health facility with in kebele	Yes	323 (57.3%)	241 (42.7%)	1	
	No	14 (31.1%)	31 (68.9%)	0.33 (0.15, 0.71)*	<0.01
Time to reach the health facility	≤30 min	109 (80.7%)	26 (19.3%)	1	
	>30 min	228 (48.1%)	246 (51.9%)	0.43 (0.23, 0.81)*	<0.01

a Adjusted odd ratio.

b Confidence interval.

c Pills and condom.

* p < 0.05.

## 4 DISCUSSION

This study sought to evaluate the awareness of obstetric fistula and the factors influencing it among women's health development armies in the Estie district, South Gondar Zone, Amhara region, Ethiopia. The results showed that 55.3% of participants (95% CI: 51.7%–57.2%) had a good understanding of obstetric fistula. Despite this level of awareness, a considerable portion of women remain uninformed about the condition. Consequently, preventing this public health issue in the community remains challenging.

This finding aligns with a study conducted in Delanta district, Amhara region, which reported a similar awareness level of 55.4%.^25^ The comparable results are likely due to both studies being carried out in the same region of Northwest Ethiopia, reflecting similar awareness levels. However, this result is higher than the 39% reported in the EDHS (2016), where two in five women surveyed had heard of obstetric fistula.^18^ This discrepancy may be attributed to the EDHS sampling different regions of Ethiopia, which vary in geography, social, and cultural factors. Additionally, this awareness level is lower than that found in studies from Nigeria and Kenya, which reported 57.8% and 60.1% respectively.^26^, ^27^ The lower awareness in our study might be related to the inclusion of only women as participants and the community‐based nature of our study, as opposed to facility‐based studies that included both men and women.

The findings of this study indicate a significant relationship between awareness of obstetric fistula and the distance to the nearest health center. Women living more than 30 min away on foot from a health center were 57% less likely to have good awareness of obstetric fistula compared to those living within 30 min. Similar studies in Ethiopia have also found that the distance to a healthcare facility impacts both awareness of obstetric fistula and health‐seeking behaviors. When health facilities are closer, women are more likely to visit them, as they do not face the challenge of transportation.^5^

Women with no history of abortion were 62% less likely to have good awareness of obstetric fistula compared to those with a history of abortion. This finding is consistent with studies conducted in Ethiopia and Burkina Faso. One reason for this difference may be that obstetric fistula is a complication often discussed during pregnancy.^5^, ^24^ The study also found that good awareness of obstetric fistula was more common among women with a history of antenatal care (ANC). Women with no pregnancy history were 48% less likely to have good awareness. This result aligns with research from the Amhara Region, which reported that women without a pregnancy history were 87% less likely to have good awareness, and with other studies showing an 80% lower likelihood of good awareness among women without pregnancy histories.^5^, ^24^ This lower awareness among women with no pregnancy history may be attributed to the fact that pregnant women are more likely to engage with maternal healthcare services, which often provide health education.^24^

Study participants with a higher number of pregnancies were more likely to be aware of obstetric fistula compared to those with fewer pregnancies. This finding is consistent with a study conducted in Ethiopia.^11^ Women with more pregnancies are more likely to access maternal health care services, which increases their exposure to information about obstetric fistula from health professionals.^11^

## 5 CONCLUSIONS AND RECOMMENDATIONS

In this study, only 55.3% of the Women's Health Development Army had good awareness of obstetric fistula, indicating a low level of awareness. Factors such as ANC follow‐up history, abortion history, pregnancy history, number of pregnancies, and proximity to a health facility were significantly associated with awareness of obstetric fistula.

Hence, local and regional health offices should design health education programs to ensure that the majority of women have optimal awareness of obstetric fistula. Given that the majority of respondents had access to television, radio, and other media, awareness programs should utilize these channels to enhance women's awareness. This study suggests placing greater emphasis on the use of basic maternal health services like antenatal care (ANC). Healthcare providers should include obstetric fistula awareness in their health education programs. Additionally, researchers are recommended to conduct qualitative studies to address perception and behavioral factors and to explore the lived experiences of women.

## 6 LIMITATIONS

Some questions were prone to recall bias, as they required women to remember past events that they might not recall accurately. To mitigate this, respondents were only asked to recall events from the past year. Additionally, being a quantitative study, this research may not fully capture the lived experiences of women with obstetric fistula, nor does it delve deeply into the cultural and behavioral factors entrenched in the community.

## AUTHOR CONTRIBUTIONS

**Bilikew Addimasu**: Conceptualization; investigation; writing—original draft; methodology; writing—review and editing; formal analysis; data curation; project administration. **Dabere Nigatu**: Conceptualization; validation; methodology; writing—review and editing; software; formal analysis; supervision. **Zemenu S. Yadita**: Conceptualization; methodology; validation; writing—review and editing; software; formal analysis; supervision. **Mekonnen Melkie**: Conceptualization; methodology; validation; writing—review and editing; software; formal analysis; supervision. All authors have reviewed and approved the final version of the manuscript.

## CONFLICT OF INTEREST STATEMENT

The authors declare no conflict of interest.

## ETHICS STATEMENT

The study was conducted following the receipt of written ethical approval from the Institutional Review Board (IRB) at the College of Medicine and Health Sciences, Bahir Dar University. Informed written consent (Supporting Information S1: File 1) was obtained from each participant. All participant information was kept strictly confidential, and their names were not recorded. Data were coded and entered into a computer protected by a personal password to ensure confidentiality.

## TRANSPARENCY STATEMENT

The lead author Zemenu Shiferaw Yadita affirms that this manuscript is an honest, accurate, and transparent account of the study being reported; that no important aspects of the study have been omitted; and that any discrepancies from the study as planned (and, if relevant, registered) have been explained.

## Supporting information

Supporting information.

Supporting information.

Supporting information.

## Data Availability

All data supporting the findings is submitted with the manuscript. The data set for this article is attached as Supporting Information S2: File 2. The corresponding author had full access to all study data and assumes complete responsibility for the data's integrity and the accuracy of the data analysis. The data that supports the findings of this study are available in the Supporting Information of this article.
