# Supplementary material for: Awareness on obstetric fistula and associated factors among women health development army, in the South Gondar zone, Amhara region, Ethiopia: A cross sectional study
Source: Health Sci Rep. 2024 Oct 14;7(10):e70141. doi: 10.1002/hsr2.70141 (PMC11473375; doi:10.1002/hsr2.70141)
Supplement: Supplementary file 1 — Supporting information. [file HSR2-7-e70141-s003.docx]

## Consent form

I have been told that this research is undertaken by Bilikew Addmasu, Dabere Nigatu Zemenu Shiferaw and Mekonnen Melkie. I have been fully informed in the language I understand and the objective of this research is to “Awareness on obstetric fistula and associated factors among women health development army, in the south Gondar zone, Amhara, Ethiopia.”

I have been also informed that all the information I provide to the interviewer will be kept confidential. I understood that the research has no any risk. I also knew that I have the right to not answer the question that I don’t want to answer or to withdraw from the study at any time I have acquainted nobody will enforce me to explain the reason of withdrawal.

I read this form, or it has been read to me in the language I understand and I understood the condition stated above.

1. Therefore, I am willing to participate ____________ (signature)
2. But, I am not willing to participate ______________ (tick)

Interviewer signature ___________________________ Date ______________________
